# Targeting DNA damage response as a potential therapeutic strategy for head and neck squamous cell carcinoma

**DOI:** 10.3389/fonc.2022.1031944

**Published:** 2022-10-21

**Authors:** Huimin Lei, Ading He, Yingying Jiang, Min Ruan, Nannan Han

**Affiliations:** ^1^ School of Stomatology, Weifang Medical University, Weifang, China; ^2^ Department of Oral Maxillofacio-Head and Neck Oncology, Shanghai Ninth People's Hospital, College of Stomatology, Shanghai Jiao Tong University School of Medicine, Shanghai, China; ^3^ Shanghai Key Laboratory of Stomatology, Shanghai Research Institute of Stomatology, National Center for Stomatology, National Clinical Research Center for Oral Diseases, Shanghai, China

**Keywords:** head and neck squamous cell carcinoma (HNSCC), DNA damage response (DDR), PARP1 inhibitor, ATR and ATM inhibitor, DNA-PK inhibitor, WEE1 inhibitor

## Abstract

Cells experience both endogenous and exogenous DNA damage daily. To maintain genome integrity and suppress tumorigenesis, individuals have evolutionarily acquired a series of repair functions, termed DNA damage response (DDR), to repair DNA damage and ensure the accurate transmission of genetic information. Defects in DNA damage repair pathways may lead to various diseases, including tumors. Accumulating evidence suggests that alterations in DDR-related genes, such as somatic or germline mutations, single nucleotide polymorphisms (SNPs), and promoter methylation, are closely related to the occurrence, development, and treatment of head and neck squamous cell carcinoma (HNSCC). Despite recent advances in surgery combined with radiotherapy, chemotherapy, or immunotherapy, there has been no substantial improvement in the survival rate of patients with HNSCC. Therefore, targeting DNA repair pathways may be a promising treatment for HNSCC. In this review, we summarized the sources of DNA damage and DNA damage repair pathways. Further, the role of DNA damage repair pathways in the development of HNSCC and the application of small molecule inhibitors targeting these pathways in the treatment of HNSCC were focused.

## Introduction

Head and neck squamous cell carcinoma (HNSCC) is the sixth most common cancer worldwide, with approximately 650,000 new cases and 350,000 deaths each year (1). Oral squamous cell carcinoma (OSCC) is the most common malignant type of HNSCC (2). Major risk factors for HNSCC include tobacco use, excessive alcohol consumption, and human papillomavirus (HPV) infection (3–5). For patients with HPV-related HNSCC, the prognosis is favorable, with an overall survival (OS) rate of 95%-80% at 2-5 years (6). However, the prognosis of patients with non-HPV and smoking-related HNSCC remains poor, with a five-year survival rate of only approximately 50% (7). Current standard treatment options for HNSCC include surgery, chemotherapy, and radiation therapy, but recurrence rates are high, and about half of all HNSCC patients experience recurrence (8). Thus, the exploration of novel and effective therapies to improve the prognosis and survival of patients with HNSCC is warranted.

It is known that DNA damage and abnormal DNA damage response (DDR) may harm the integrity and stability of the whole genome and contribute to various diseases such as cancer (9). To maintain genome integrity and suppress tumorigenesis, individuals have acquired a series of repair functions, termed the DDR, during evolution to repair DNA damage and ensure the accurate transmission of genetic information. DDR is a complex kinase-based signaling pathway that senses, transduces, and responds appropriately to DNA damage to maintain genomic stability (10). Mutations in any component of DDR are considered to be related to the initiation and progression of as breast cancer (11), ovarian cancer (12), prostate cancer (13), colorectal cancer (14) and HNSCC (15), which have been previously reported. Therefore, targeting the DDR pathway may be a novel therapeutic approach for HNSCC treatment. This paper comprehensively reviewed the various reported sources of DNA damage and associated repair pathways in HNSCC and drugs targeting the abnormal DNA damage response pathway.

## DNA damage repair pathway

It is estimated that cells experience thousands of DNA damage events daily, which can be divided into two broad categories based on their origin: endogenous and exogenous (16, 17). Most endogenous DNA damage arises from the hydrolysis and oxidation of chemically active DNA with water and naturally occurring intracellular reactive oxygen species (ROS) (18, 19). On the other hand, exogenous DNA damage is caused by environmental, physical, and chemical agents such as ultraviolet (UV) and ionizing radiation (IR), alkylating agents, and crosslinking agents (16). These agents can induce different types of DNA damage, such as abasic sites, mismatches, interstrand crosslinks, or single- and double-stranded breaks (19). The most harmful lesions and the most serious threat to cells are double-strand breaks (DSBs). If repaired ineffectively or incorrectly, DSBs can lead to carcinogenesis or cell death (20). Different types of DNA damage are identified and repaired by the corresponding DDR pathways, and these repair processes are key to maintaining the genetic stability of the cells.

**Figure 1:**
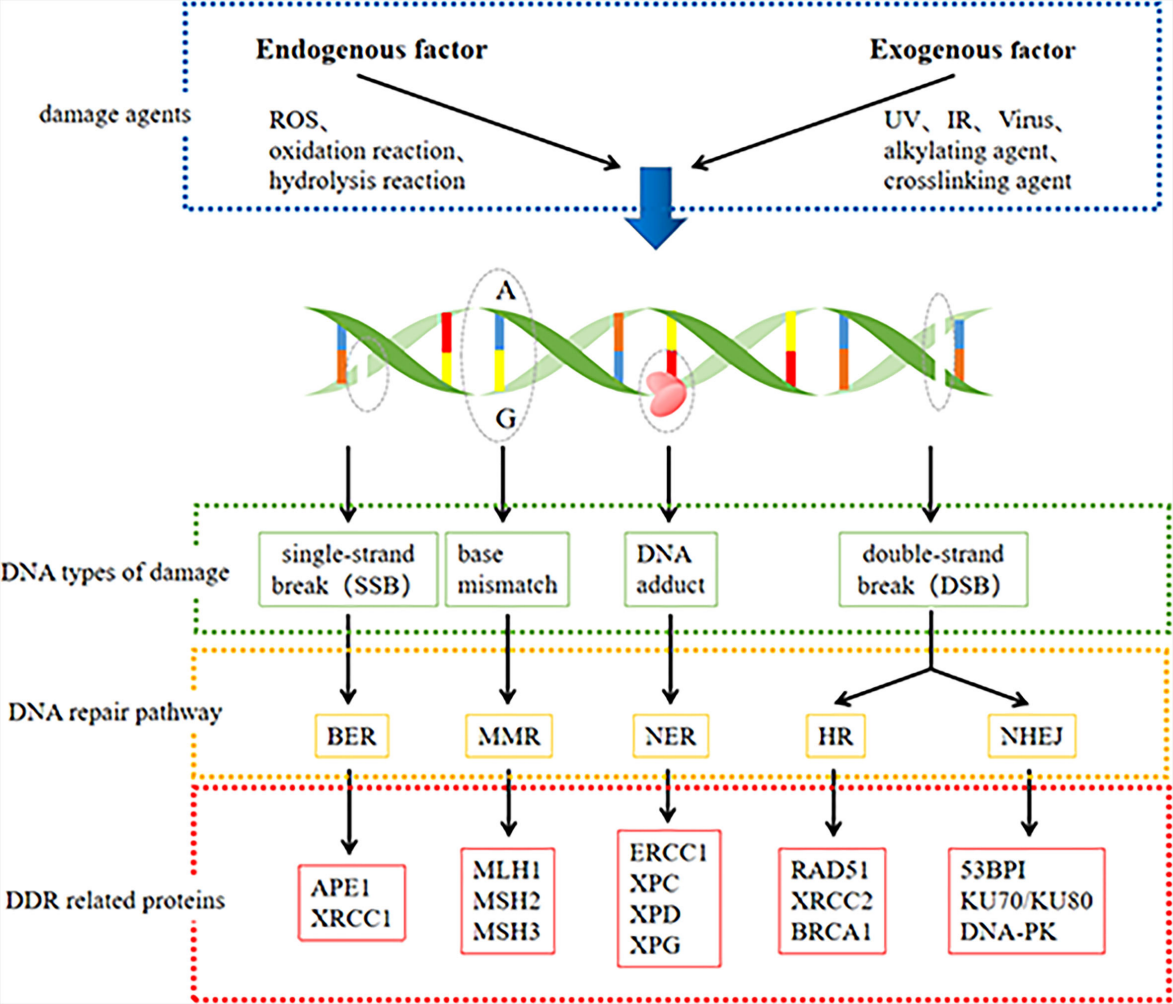
DNA damage and main DNA repair pathway and some associated protein.

DDR pathways include mismatch repair (MMR), base excision repair (BER), nucleotide excision repair (NER), homologous recombination (HR), and non-homologous terminal link (NHEJ) repair (21). A few specific lesions can also be removed using direct reversal repair (DR) pathways and interstrand crosslink (ICL) repair (16). These repair processes are key to maintaining the genetic stability of cells (22). We summarized the DNA damage and main DDR pathways and some associated proteins in **Figure 1**. The BER pathway repairs base lesions caused by oxidation, deamination, and alkylation (23). NER mainly removes bulky DNA adducts induced by UV radiation or chemotherapeutic drugs (24). The MMR system is responsible for correcting base-base mismatches and insertion or deletion mismatches generated during DNA replication (25). DSBs are the most severe type of DNA damage and can be repaired by HR, NHEJ, or both repair pathways (26). The HR pathway facilitates highly accurate DSB repair by utilizing homologous DNA sequences on the sister chromatid as a replication template during repair, while the NHEJ pathway does not require a homologous template and is highly efficient but intrinsically error-prone (18). ICL repair involves a complex interplay between multiple DNA repair pathways, including NER, HR, Fanconi anemia (FA), and translesion DNA synthesis (TLS) (27).

DDR involves the detection of DNA damage, activation of cell cycle checkpoints, induction and recruitment of repair factors to the damage sites, and the subsequent repair of the damage. Effective damage repair results in cell cycle resumption, whereas improper repair or damage beyond repair results in permanent cell cycle arrest (senescence) or programmed cell death (apoptosis). Failure to efficiently repair DNA damage can cause mutations and genome instability that drive cancer (19). Defective DDR pathways are a common characteristic of cancer. Alterations in DDR-related genes, such as somatic or germline mutations, single nucleotide polymorphisms(SNPs) and promoter methylation of key genes, and other epigenetic alterations, are closely related to the occurrence and development of cancer (17). Understanding the internal mechanism and correlations between DDR pathway alterations and cancer can improve the efficacy of anti-tumor therapy and play a role in prediction and prognosis.

In this review, we discuss recent progress in the research area of targeted DDR pathways for HNSCC.

## DNA damage repair in HNSCC

It has been shown that germline and somatic alterations in DNA repair genes are associated with different susceptibility and prognosis in patients with HNSCC. In a retrospective study of 170 patients with HNSCC, BRCA2 and ARID1A were found to be the two DDR genes with the highest mutation rates, with 17.6% of patients having mutations in these two genes, followed by ATM and BRCA1 with 13.5% and 10% mutations, respectively (28). In addition, a recent circulating tumor (ctDNA) sequencing study of 75 patients with HNSCC found that the five most commonly altered genes were TP53, CDKN2A, TERT, BRCA2, and NOTCH1, and that 38.8% of patients had alterations in DNA repair genes (APC, ATM, BRCA1, or BRCA2) (29). For the first time, ctDNA alterations in TP53 and DNA repair genes were shown to be significantly associated with poor prognosis in HNSCC (29).

Another whole-exome sequencing study of 45 patients with oral and oropharyngeal cancer found that FANCG, CDKN2A, and TPP germline variants were strongly associated with HNSCC risk (30). At least one germline variation in DNA repair pathway genes was detected in 67% of patients (30). This study found that young adults with germline variants of the DNA repair gene had an increased risk of HNSCC susceptibility, while patients with germline variants of the DNA repair gene also had a higher five-year survival rate (30). Moreover, a high frequency of somatic alterations in TP53, CDKN2A, FAT1, and PIK3CA was confirmed in 521 patients with TCGA-HNSCC (30). TP53 has a central node in the interaction network between somatic and germline mutant genes (30). In addition, several significantly altered genomic features were found in OSCC patients with lymph node metastasis (LNM) compared with patients without LNM, including hotspot somatic mutations in TP53 and CASP8, rare nonsilent germline mutations in BRCA2 and FAT1, mutations in mitotic G2/M and NHEJ pathways, recurrent deletions of DNA repair genes by homologous recombination, and chromosomal instability (31). Furthermore, these characteristic changes were of high predictive value for LN metastasis, and LN+ patients with mutations in the NHEJ pathway had longer disease-free survival (31).

Abnormal expression of DDR-related genes is associated with the development, progression and treatment response of HNSCC (**Table 1**). In a study of 70 HNSCC patients and 46 healthy controls (HC), DDR-related parameters were disrupted in HNSCC patients. NER) (ERCC1, ERCC2/XPD, XPA, and XPC) and BER (APEX1, XRCC1) genes were downregulated in patients with HNSCC compared with the HC, whereas double-strand break repair (MRE11A, RAD50, RAD51, XRCC2) and mismatch repair (MLH1, MSH2, MSH3) genes were up-regulated (15). A study of NER gene expression levels in the peripheral blood lymphocytes of 483 subjects (251 HNSCC patients and 232 HC) found that XPA and XPB expression levels in HNSCC patients were significantly lower than those in the controls, and lower XPB gene expression levels were associated with an increased risk of HNSCC (35). In a study of 349 newly diagnosed HNSCC patients and 295 cancer-free controls, ERCC3 and XPA expression levels were significantly reduced in HNSCC patients compared with controls (36). Further analysis revealed a dose-dependent relationship between the increased risk of HNSCC and low ERCC3 and XPA expression levels (36). Further studies have shown that a lower NER capacity is associated with longer progression-free survival (15). A study of NER core proteins (ERCC1, XPF, and XPA) in 453 HNSCC patients found that high expression of ERCC1 and XPA was associated with poor OS in patients with OSCC, which may be related to chemotherapy resistance (32). However, in oropharyngeal SCC, high XPA expression was associated with a significantly better OS (32). Studies have showed that oropharyngeal SCCa patients with high ERCC1 expression may have better outcomes, independent of HPV status (49). However, it has also been reported that ERCC1 expression is not associated with the prognosis of oropharyngeal/oral SCCHN (50). In addition, polymorphisms of NER core genes (XPC, XPD and XPG) have been shown to be associated with the recurrence of SCCOP (51). The relationship between the expression of NER related genes and the prognosis of oropharyngeal carcinoma needs to be further studied. The differential expression of NER core protein in the head and neck subsites affects the OS of patients, which is of great significance for overcoming chemotherapy resistance in the HNSCC subtype (32). Moreover, studies have reported that high ERCC1 expression is significantly associated with enhanced chemotherapy response induced by 5-FU and cisplatin, suggesting that ERCC1 expression can be used as a biomarker to predict the efficacy of 5-FU and cisplatin chemotherapy for HNSCC (33). Conversely, another study showed that high ERCC1 expression was associated with cisplatin resistance and poor prognosis, while low ERCC1 expression was more sensitive to cisplatin in HNSCC (34). Meanwhile, previous studies have shown that the expression of NER protein (ERCC1, ERCC2) may be sensitive prognostic indicators of radiochemotherapy for locally advanced HNSCC (52–54). In addition, polymorphisms of ERCC1, ERCC2(XPD), XPA and XPC have been found to be associated with radiotherapy efficacy in a variety of tumors, including HNSCC, and may be important predictors of clinical outcomes in patients treated with radiotherapy (55–60). Studies have found that the expression of NER related genes and single nucleotide polymorphisms (SNPs) may also influence the severity of radiation-related side effects in patients (61–64). Thus, NER proteins is not only closely related to the occurrence of tumors, but it also is a predictor for the efficacy of radiotherapy and prognosis of tumors. In oral tongue squamous cell carcinoma (OTSCC), the expression of the proteins APE-1 and XRCC-1 (involved in BER) was found to have increased, and XRCC-1 expression was correlated with better clinical staging of OTSCC and negative lymph node metastasis (38). This suggests that this protein may play a protective role (38). In addition, previous studies have shown that APEX1 expression levels in HNC tumor tissues are significantly higher than those in normal tissues, and that high APEX1 expression is associated with an increased risk of HNC in the Pakistani population (37). The proteins MLH1 and MSH2 (involved in MMR) are overexpressed in oral leukoplakia with dysplasia and OSCC, especially in patients with comorbidities (39). In contrast, other studies have shown that the expression of MMR repair-associated genes MLH1, MSH2, MLH3, and PMS2 is decreased in OSCC (40). Low expression of these genes is associated with reduced DNA repair capacity and development of malignant lesions (40). Additionally, previous studies have reported that reduced expression of these genes is correlated with smoking-induced methylation in smokers at risk of cancer (41). DSBs are mainly repaired by the HR and NHEJ pathways. High expression of DSB repair-related genes (MRE11A, Rad50, RAD51, and XRCC2) in HNSCC patients may contribute to tumor acquisition and progression or induce drug-resistant phenotypes (15). A recent study also showed that high RAD51 expression was associated with the occurrence, development, and recurrence of OSCC, indicating a poor prognosis (43). Moreover, studies have shown that ATM and BRCA1 (both involved in HR) are lowly expressed in HNSCC and that low expression of both is associated with decreased OS and poor prognosis in HNSCC patients (44, 45). Mre11 (part of the MRN complex) is essential for DSB repair in normal cells. Studies have shown that the expression of Mre11 in oral cancer tissues is significantly higher than that in adjacent non-cancerous oral tissues and that oral cancers with high MRE11 expression have reduced OS and progression-free survival (42). In addition, the mediator of DNA damage checkpoint protein 1 (MDC1) is associated with the recruitment of DNA damage repair proteins to the site of DSBs (65). Clinical studies have shown that in OSCC (100 patients), nuclear and cytoplasmic MDC1 protein expression levels were 85% and 92%, respectively. Strong nuclear expression of MDC1 was significantly associated with lymph node metastasis and reduced relapse-free survival (RFS) in patients with OSCC. By contrast, patients with weak nuclear MDC1 expression benefited significantly from radiation therapy after surgery (66). Therefore, high expression of MDC1 correlates with the aggressiveness of OSCC and can serve as an important prognostic indicator for OSCC patients (66). The expression level of KU70/80, a protein involved in the NHEJ repair pathway, was higher in OSCC tissues than in normal tissues, and its high expression was associated with shorter OS of patients (46). In addition, Ku80 expression levels were associated with higher local recurrence rates after radiotherapy in patients, suggesting that Ku80 may be a potential prognostic biomarker for HNSCC patients (47). The expression of DNA-PKcs in HPV-positive oropharyngeal squamous cell carcinoma tissues was significantly lower than that in HPV-negative oropharyngeal squamous cell carcinoma tissues, and the expression of DNA-PKcs negatively correlated with the expression of HPV E6 and E7 (48). DNA-PKcs knockdown leads to an increased sensitivity of tumor cells to cisplatin and radiotherapy, and reduces cell migration and invasion (48).

**Table 1:** DDR pathway alterations in HNSCC.

DNA damage pathway	Genes/proteins	Alternation in HNSCC (expression)	Prognosis	References
NER	ERCC1	low	related to longer progression-free survival in HNSCC	(15)
		high	related to poor OS in patients with OSCC	(32)
			related to enhanced chemotherapy response induced by 5-FU/cisplatin in HNSCC	(33)
			related to cisplatin resistance and poor prognosis of HNSCC	(34)
	XPB	low	related to an increased risk of HNSCC	(35, 36)
	XPA	low	related to an increased risk of HNSCC	(36)
			related to longer progression-free survival in HNSCC	(15)
		high	related to poor OS in patients with OSCC	(32)
			related to a significantly better OS in oropharyngeal SCC	(32)
BER	APEX1	high	related to an increased risk of HNSCC and positive lymph metastasis in the Pakistani population	(37)
	XRCC1	high	related to better clinical staging of OTSCC and negative lymph node metastasis	(38)
MMR	MLH1	high	related to increased risk of OSCC, especially in patients with comorbidities	(39)
		low	related to reduced DNA repair capacity and the development of malignant lesions	(40)
			related to smoking-induced methylation in smokers at risk for cancer	(41)
	MLH2	high	related to increased risk of OSCC, especially in patients with comorbidities	(39)
		low	related to reduced DNA repair capacity and the development of malignant lesions	(40)
			related to smoking-induced methylation in smokers at risk for cancer	(41)
	MSH3	low	related to reduced DNA repair capacity and the development of malignant lesions	(40)
			related to smoking-induced methylation in smokers at risk for cancer	(41)
HR	MRE11A	high	related to tumor acquisition and progression and/or induce drug-resistant phenotypes	(15)
			related to reduced overall survival and progression-free survival	(42)
	RAD51	high	related to tumor acquisition and progression and/or induce drug-resistant phenotypes	(15)
			related to the occurrence, development and recurrence of OSCC, indicating a poor prognosis	(43)
	BRCA1	low	related to decreased OS and poor prognosis in HNSCC patients	(44, 45)
NHEJ	ku70/ku80	high	related to reduced OS of OSCC patients	(46)
			related to higher local recurrence rates after radiotherapy in HNSCC patients	(47)
	DNA-PKcs	low	related to increased therapeutic sensitivity to cisplatin and radiotherapy in oropharyngeal SCC	(48)

SNPs in several DDR and DNA repair-related genes have been reported to be associated with an increased risk of HNSCC (**Table 2**), including ERCC1 (67), ERCC2/XPD (67–70), XPC (71, 72) and XPG (69) (involved in NER), APEX1 (73) and XRCC1 (74–76)(involved in BER), MLH1 (77), MSH2 (77, 78), MSH3 (77, 79) and EXO1 (77, 80) (involved in MMR), XRCC3 (70, 76, 81, 83, 84) and RAD51 (81–84, 90) (involved in HR), Ku70 (85)/80 (86, 87), XRCC4 (88, 89) and Lig4 (83)(involved in HR). Studies on these gene polymorphisms have potential benefits in identifying predictive biomarkers for the risk of HNSCC and provide useful evidence for the early detection of HNSCC. However, comprehensive functional investigations of DDR-related gene SNPs are still lacking. Therefore, further research on SNPs and risk alleles is required.

**Table 2:** SNPs and DDR related genes mutations in HNSCC.

Pathway	Gene	Polymorphism	Significance	Reference
NER	ERCC1	rs11615	reduced risk	(67)
	XPD/ERCC2	rs1799793	increased risk	(68)
		rs13181	increased risk	(68)
			reduced risk	(67, 69, 70)
	XPG	rs17655	increased risk	(69)
	XPC	rs2228001	increased risk	(71)
		PAT	increased risk	(72)
		Ala499Val	increased risk	(72)
BER	APEX1	Asp148Glu	increased risk	(73)
	XRCC1	Arg399Gln	increased risk	(74–76)
		Arg194Trp	increased risk	(74)
MMR	MLH1	rs1800734	increased risk	(77)
	MSH2	rs2303426	increased risk	(77)
		gIVS12-6C	reduced risk	(78)
	MSH3	rs26279	increased risk	(77)
		rs12515548	increased risk	(79)
	EXO1	rs1047840	increased risk	(77, 80)
HR	RAD51	G135C	increased risk	(81, 82)
		rs1801320	reduced risk	(83)
		rs5030789	reduced risk	(84)
		rs1801321	reduced risk	(84)
	XRCC3	rs3212057	increased risk	(84)
		rs861539/Thr241 Met	increased risk	(70, 81, 83)
NHEJ	Ku70	rs5751129	increased risk	(85)
	Ku80	A2790G	increased risk	(86)
		rs828907	increased risk	(87)
	XRCC4	rs28360071	increased risk	(88)
		rs3734091	increased risk	(89)
	Lig4	rs1805388	reduced risk	(83)

Additionally, epigenetic alterations have been reported to be closely related to the development and progression of cancer. A meta-analysis has suggested that hypermethylation of the MLH1 promoter is associated with HNSCC. Thus, methylated MLH1 could be a potential diagnostic biomarker for HNSCC (91). One study showed that epigenetic silencing of O6-methylguanine-DNA methyltransferase (MGMT, involved in the DR pathway) DNA repair enzyme through promoter hypermethylation (HmMGMT) may increase TP53 oncosuppressor gene mutations, thereby promoting HNSCC (92). In addition, patients with both hmMGMT and destructive TP53-mutations may have a poor prognosis (92). Hypermethylation of the DNA glycosylase NEi endonuclease VIII-like 1 (NEIL1) promoter has been demonstrated in HNSCC, with increased methylation levels in tumors compared to matched non-tumor cells. DNA methylation contributes to the downregulation of NEIL1, which is involved in the BER repair pathway, thereby increasing the sensitivity of HNSCC to chemotherapy or radiotherapy (93). Another study reported that methylation loss at the three-prime repair exonuclease 2 (TREX2) locus was observed in laryngeal cancer, and that low TREX2 DNA methylation was associated with elevated TREX2 expression and prolonged OS in laryngeal cancer (94).

In summary, these data show that DNA damage and aberrant DDR play an important role in the occurrence, development, treatment response, and prognosis of HNSCC (95).

## New therapeutic approaches targeting the DDR pathway in HNSCC

### PARP inhibitors

PARPs are a group of enzymes that utilize beta nicotinamide adenine dinucleotide (β-NAD+) to covalently add Poly(ADPribose) (PAR) chains onto target proteins, a process known as PARylation (96). The most studied and best understood is PARP1, which is the first member of the poly ADP-ribosyl polymerase (PARP) superfamily (97). PARP1 plays an important role in DNA damage repair and the maintenance of genome integrity (98). It binds to nuclear DNA single-strand break (SSB) and recruits a number of different DNA repair proteins, such as x-ray repair cross complimenting protein 1 (XRCC1), to repair the SSB (99). PARP1 is also involved in several other forms of DNA repair, including BER, NER, HR, NHEJ, and mismatch repair (99). Hence, inhibition of PARP1 and the related enzyme PARP2 could be a better therapeutic approach to target specific DNA repair pathways in cancer (100). Actually, PARP inhibitors have proven significant clinical benefits in a variety of solid tumors, including HNSCC (95, 101). Several PARP inhibitors, including olaparib, have been approved by the FDA for the treatment of cancers, including breast, ovarian, prostate and pancreatic cancers. Thus, we summarizes the application of DDR inhibitors in pre-clinical or reported clinical therapies combination with other anticancer therapies for HNSCC (**Table 3**). Moreover, the relevant registered clinical trials of DDR inhibitors, alone or combined with other therapies in HNSCC, are summarized in **Table 4**.

**Table 3:** DDR inhibitors in pre-clinical or reported clinical therapies combination with other anticancer therapies for HNSCC.

Target	Inhibitor	Intervention	Phase	Efficacy	Reference
PARP	Olaparib	Olaparib+RT	Pre-clinical trials	Enhances the radiosensitivity of OPSCC, particularly for HPV-negative OPSCC	(102)
		Olaparib+NU7441+RT	Pre-clinical trials	Enhances HPV-negative HNSCC inhibition *in vitro* and vivo	(103)
		Olaparib+adavosertib/prexasertib+RT	Pre-clinical trials	Results in highly effective radiosensitization of HPV-positive HNSCC	(104)
		Olaparib/veliparib+cisplatin/5-fluorouracil	Pre-clinical trials	reverses the treatment resistance and sensitizes the tumor response	(105)
		Olaparib ± RT	Phase I	Enhances therapeutic response in SMAD4-deficient HNSCC	(106)
		Olaparib+cetuximab+RT	Phase I	Improves outcomes and reduces dermatitis in locally advanced HNSCC with heaving smoking histories	(107)
		Olaparib+cisplatin+RT	Phase I	Improves the therapeutic effect and minimizes treatment associated toxicity	(108)
	Veliparib	Veliparib+carboplatin+paclitaxel	Phase I	Improves the survival rate in advanced HNSCC patients and has a good safety	(109)
	Niraparib	Niraparib+RT	Pre-clinical trials	Enhances the sensitivity of HNSCC cells to photon and proton	(110)
		Niraparib+RT	Pre-clinical trials	Enhances the radiosensitivity of HNSCC cells, especially in HPV-negative HNSCC cells	(111)
		Niraparib+MK-8776+RT	Pre-clinical trials	Enhances the radiosensitivity of HPV-positive HNSCC cells	(111)
		Niraparib+MK-1775	Pre-clinical trials	Enhances the radiosensitivity of HPV-negative HNSCC cells	(111)
ATR	AZD6738	AZD6738+cisplatin	Pre-clinical trials	Enhances the sensitivity of HPV-negative and HPV-positive HNSCC cells to cisplatin	(112)
		AZD6738+RT	Pre-clinical trials	Enhances the sensitivity of HNSCC cells to radiotherapy, independent of HPV status	(113)
	VE-822	VE-822+RT	Pre-clinical trials	Shows a synergistic effect in inhibiting tumor growth of HNSCC	(114)
ATM	AZD0156	AZD0156+RT	Pre-clinical trials	Shows a synergistic effect in inhibiting tumor growth of HNSCC	(114)
	KU55933	KU55933+cisplatin	Pre-clinical trials	Potentiates cisplatin-induced cytotoxicity in HNSCC cells	(45)
	GSK63541A	GSK63541A+RT	Pre-clinical trials	Shows highly selective radiosensitization activity in HNSCC cells	(115)
DNA-PK	AZD7648	AZD7648+RT	Pre-clinical trials	Enhances the radiosensitivity of HNSCC cells	(116)
	KU-57788	KU-57788+RT	Pre-clinical trials	Enhances the radiosensitivity of HNSCC cells, especially in HPV-negative HNSCC cells	(117)
	KU-0060648	KU-0060648+AZD6738+RT	Pre-clinical trials	Enhances the radiosensitivity of HNSCC cells, independent of the P53 status	(118)
	CC-115	CC-115	Phase I	Shows good safety and preliminary efficacy in HNSCC	(119)
WEE1	AZD1775	AZD1775+ricolinostat	Pre-clinical trials	Shows synergistic cytotoxicity in HNSCC cells with TP53 mutations or impaired P53 function	(120)
		AZD1775+LY2606268	Pre-clinical trials	Shows high cytotoxicity against HPV-negative HNSCC cells with TP53 mutations	(121)
		AZD1775+LY2603618/MK8776+RT	Pre-clinical trials	Exhibits radiosensitization effect in HPV-positive HNSCC cells	(122)
		AZD1775+RT	Pre-clinical trials	Enhances radiotherapy effects in HNSCC xenografts	(123)
		AZD1775+BO2	Pre-clinical trials	Significantly inhibits tumor growth in HPV-positive HNSCC xenografts	(124)
		AZD1775+MLN8237	Pre-clinical trials	Shows synergistic anticancer effects in HNSCC cells	(125)
		AZD1775+cisplatin	Pre-clinical trials	Improves the efficacy of cisplatin resistant HNSCC	(126)
		AZD1775+cisplatin+docetaxel	Phase I	Shows synergistic anti-tumor effect in advanced HNSCC patients	(127)
		AZD1775+cisplatin+RT	Phase I	Enhances the radiosensitivity and shows good safety in TP53 mutant HNSCC patients	(128, 129)

**Table 4:** Clinical trials of DDR inhibitors alone or combined with other treatments in HNSCC.

Target	Inhibitor	NCT Number	Additional Treatments	Phase	N	Status
PARP	Olaparib	NCT02308072	Cisplatin, IMRT	I	70	Active, not recruiting
		NCT02229656	Radiotherapy	I	12	Active, not recruiting
		NCT01758731	Cetuximab, Radiation Therapy	I	17	Completed
		NCT02882308	Cisplatin, Durvalumab	II	41	Completed
		NCT05366166	Pembrolizumab, Cisplatin, IMRT	II	45	Not yet recruiting
		NCT03022409	Ceralasertib	I	21	Completed
		NCT04643379	Pembrolizumab, Carboplatin	II	30	Recruiting
		NCT04825990	Pembrolizumab	II	30	Recruiting
		NCT03085147	–	I/II	39	Recruiting
	Niraparib	NCT04313504	Dostarlimab	II	23	Recruiting
		NCT05169437	–	II	110	Recruiting
		NCT04779151	Dostarlimab	II	112	Not yet recruiting
		NCT04681469	–	II	49	Recruiting
		NCT05162872	Sintilimab	II	99	Recruiting
		NCT03088059	Carboplatin, Durvalumab, Afatinib, Palbociclib, IPH2201 BAY1163877 MTX, Paclitaxel, Docetaxel, 5-FU, Bleomycin, Gemcitabine, Mitomycin	II	340	Recruiting
	Veliparib	NCT01711541	Carboplatin, Cisplatin Fluorouracil, Hydroxyurea Paclitaxel, Radiation Therapy	I/II	24	Active, not recruiting
		NCT01366144	Carboplatin, Paclitaxel	I	94	Active, not recruiting
	Talazoparib	NCT04052204	avelumab, Bempegaldesleukin, talazoparib, enzalutamide	I/II	3	Terminated
ATR	BAY 1895344	NCT04576091	Elimusertib, Pembrolizumab, Radiation Therapy	I	37	Recruiting
		NCT04491942	Cisplatin, Elimusertib, Gemcitabine Hydrochloride	I	74	Recruiting
	M6620	NCT02567422	Cisplatin, Radiation Therapy	I	45	Active, not recruiting
DNA-PK	M3814	NCT04533750	IMRT	I	42	Recruiting
	CC-115	NCT01353625	–	I	118	Completed
WEE1	MK-1775	NCT02196168	Cisplatin	II	6	Terminated
		NCT03028766	Cisplatin, Radiotherapy	I	9	Completed
		NCT02508246	Cisplatin, Docetaxel Therapeutic Surgery	I	12	Completed
		NCT02585973	Cisplatin, IMRT	I	12	Completed

IMRT, intensity modulated radiation therapy.

#### Olaparib

One study showed that the PARP inhibitor olaparib enhanced the apoptotic potential of curcumin by increasing DNA damage in oral cancer cells through inhibition of the BER cascade (100). The combination of the PARP inhibitor olaparib and radiotherapy enhances the radiosensitivity of OPSCC cells, particularly HPV-negative OPSCC cells (102). Similarly, studies have shown that the combination of the PARP inhibitor olaparib and DNA-PK inhibitor NU7441 with IR enhances HPV-negative HNSCC inhibition in both cell cultures and mice (103). In addition, the combination of the PARP inhibitor olaparib and Wee1/Chk1 inhibitor is a highly effective approach for radiosensitization of HPV-positive HNSCC cells (104). Olaparib alone or in combination with radiotherapy caused more DNA damage-associated cell death and reduced proliferation of SMAD4 deficient HNSCC cells (106). In addition, further phase I clinical trials have shown that olaparib in combination with radiotherapy enhances the therapeutic response of HNSCCs (106). A phase I trial demonstrated that olaparib combined with cetuximab and radiotherapy was well tolerated with reduced dermatitis within the radiation field in patients with locally advanced HNSCC with high-risk smoking-related tumors (107). Furthermore, phase 1 trials assessing the safety and tolerability of olaparib in combination with cisplatin and radiotherapy treatment regimens showed that the addition of olaparib improved the therapeutic effect and minimized treatment-associated toxicity to normal tissues (108). Inhibition of PARP, using olaparib or veliparib, partially reversed the resistance caused by cisplatin and 5-FU treatment, and the combination of PARP inhibitors and cisplatin and 5-FU significantly sensitized the tumor response (105).

#### Veliparib

Additional studies have shown that the PARP inhibitor veliparib (ABT-888) enhances the anti-angiogenic potential of curcumin through deregulation of NECTIN-4 in oral cancer (130). A recent phase I clinical trial demonstrated that veliparib combined with carboplatin and paclitaxel induction chemotherapy was well tolerated and improved the survival rate in patients with locoregionally advanced HNSCC (109). This indicates that combined induction therapy is safe and feasible.

#### Niraparib

A recent study reported that niraparib enhances the sensitivity of HNSCC cells to both photons and protons and increases the relative biological effectiveness of protons (110). This suggests that niraparib can improve the efficacy of photon and proton radiotherapy in patients with HNSCC patients (110). Niraparib effectively enhanced the radiosensitivity of HNSCCs, particularly HPV-negative HNSCC (111). The combination of niraparib and the Chk1 inhibitor MK-8776 further enhanced the radiosensitivity of HPV-positive HNSCC cells, whereas the combination of niraparib and the Wee1 inhibitor MK-1775 significantly increased the radiosensitivity of HPV-negative HNSCC cells (111).

Meanwhile, the combined application of PARP1 inhibitors and immunotherapy especially PD-1/PD-L1 inhibitors has also attracted much attention in recent years. It has been reported that defects in the DDR pathway may serve as predictive biomarkers of responsiveness to ICIs (131, 132). It has been found that patients with enrich DDR-related genes mutations exhibited higher response to ICIs therapy and was associated with prolonged progression-free survival (133, 134). Besides, a large number of clinical trials have demonstrated that the combination of DDR inhibitors and immune checkpoint inhibitors (ICIs) had synergistic benefit and was well tolerated, which had been observed in different types of solid tumors, including head and neck cancer (95), breast cancer (135), ovarian cancer (136), prostate cancer (137), small cell lung cancer (SCLC) (138), non-small cell lung cancer (NSCLC) (139) and colorectal cancer (140). Thus, we summarize the current clinical studies of PARP1 inhibitors in combination with PD-1/PD-L1 inhibitors as **Supplementary Table 1**.

### ATR and ATM inhibitors

Ataxia telangiectasia and RAD3-related protein (ATR) and ataxia telangiectasia mutated protein (ATM), both members of the phosphatidylinositol 3-kinase-related kinase (PIKK) family, are key sensors of the DDR signaling pathway (141). ATM is activated in response to DSBs, whereas ATR is primarily triggered by replication stress and SSBs. Activation of ATM or ATR phosphorylates its downstream targets, Chk2 or Chk1 kinase, to induce cell cycle arrest and facilitate DNA repair (141). One study showed that the ATR inhibitor AZD6738 enhanced the sensitivity of HPV-negative and HPV-positive HNSCC cells and tumors to cisplatin (112). Recent studies have found that the ATR inhibitor AZD6738 enhances the sensitivity of HNSCC to radiotherapy independent of HPV status (113). The radiosensitizing effect of AZD6738 correlated with checkpoint kinase1 (CHK1)-mediated abrogation of G2/M-arrest (113). Another key mechanism by which ATM and ATR inhibitors selectively enhance tumor cell inactivation when combined with IR) may be through the inducing of senescence (142). A recent study showed that the ATM inhibitor KU55933 induces apoptotic signaling and potentiates cisplatin-induced cytotoxicity (45). GSK63541A, a novel ATM inhibitor, has been shown to have a highly selective radiosensitization activity that is superior to cisplatin and cetuximab (115). Notably, it exhibited virtually no cytotoxicity in normal cells (115). Consistent with this, a recent study reported that ATM and ATR inhibitors in combination with IR increased the death of HNSCC cells, while the combination therapy did not increase side effects (114).

### DNA-PK inhibitors

While ATRi and ATMi have been shown to disturb cell cycle arrest and repair DSBs, cancer cells may resort to alternative repair mechanisms, such as DNA-PK-mediated NHEJ (D-NHEJ) (118). Therefore, inhibition of DNA-PK, another member of the PIKK family, may be a promising treatment method to block the DDR pathway in cancer cells after radiotherapy. A recent study suggested that DNA-PKcs inhibition in combination with radiation enhanced the radiosensitization of HNSCC cells to photons and protons and, in particular, inhibited the growth of relatively radioresistant HPV-negative HNSCC cells (117). In addition, studies have shown that the DNA-PK inhibitors KU57788 and IC87361 are more potent radiosensitizing agents than the PARP-1 inhibitors veliparib and olaparib at non-cytotoxic concentrations in HNSCC cell cultures, and that their activities can be enhanced by SLFN11 and hypoxia (143). Similarly, another study found that the DNA-PKi AZD7648 is a potent radiosensitizer in both human (UT-SCC-54C) and murine (SCCVII) HNSCC cells (116). Interestingly, the combination of ATR and DNA-PK inhibitors was more effective in increasing radiotherapy sensitivity, independent of the P53 status of HNSCC cells (118). A phase I trial demonstrated that CC-115, a dual inhibitor of mTOR kinase and DNA-PK, showed good safety and preliminary efficacy and could be a promising novel anticancer treatment agent (119).

### WEE1 inhibitors

HPV-negative HNSCC has a poor prognosis. The most common mutational events in HPV-negative HNSCC are inactivation of the tumor suppressor genes TP53 (>85%) and CDKN2A (>57%), which significantly impair G1/S checkpoints, leading to dependence on other cell cycle checkpoints to repair ongoing replication damage (121). Wee1 is a serine/threonine kinase that regulates DNA damage-induced G2/M phase arrest by phosphorylation of cyclin-dependent protein kinase 1 (CDK1, also known as CDC2) at Tyr15 residues (144). Inhibition of Wee1 kinase activity abrogates the G2/M checkpoint and induces replication stress and DNA damage, leading to unrestrained or premature mitosis and subsequent cell death through a mitotic disaster (145).

Thus, WEE1 inhibitors may be a promising approach for treating HPV-negative HNSCC and other TP53 mutated cancers (121). One study showed that Wee1 inhibition (AZD1775) was also highly sensitive to head and neck precancerous cell lines, whereas normal keratinocytes were tolerant to the inhibitor. Therefore, Wee1 inhibition therapy could be used as an effective treatment for precancerous lesions to prevent cancer (146). In one study, a combination of the Wee1 inhibitor adavosertib (AZD1775) and ricolinostat, a selective inhibitor of histone deacetylase 6 (HDAC6), showed synergistic cytotoxicity in HNSCC with TP53 mutations or impaired p53 function caused by HPV infection, possibly by enhancing adavosertib-induced mitotic disaster (120). A recent study showed that adavosertib (AZD1775), in combination with the Chk1 inhibitor prexasertib (LY2606268), showed high cytotoxicity against HPV-negative HNSCC with a compromised G1/S checkpoint caused by TP53 mutations (121). In addition, the combined inhibition of Wee1 and Chk1 efficiently abrogated the G2-arrest induced by radiation and had a radiosensitizing effect on HPV-positive HNSCC cells (122). Moreover, Wee1 inhibitors (Wee1i), in combination with radiotherapy, enhanced the effects in HNSCC xenografts (123). Studies have shown that HPV-positive HNSCCs exhibit high FOXM1 activity, and Wee1i enhances sensitivity to HPV-positive HNSCC through the CDK1-FOXM1 pathway, which drives premature mitosis and DNA damage (147). Another study reported that the combination of adavosertib (AZD1775) and a Rad51 inhibitor (B02) significantly inhibited tumor growth in a xenograft mouse model carrying HPV-positive HNSCC, compared to HPV-negative HNSCC (124). The synergies between the two drugs are associated with excessive DNA damage and replication stress induced by the forced activation of CDK1 and reduction of Chk1 phosphorylation, ultimately leading to abnormal mitosis and apoptosis (124). In addition, studies have demonstrated that adavosertib (AZD1775) enhanced the anticancer effects of the AURKA inhibitor alisertib (MLN8237) in HNSCC, both *in vitro* and *in vivo* (125). The Wee1i AZD1775 combined with cisplatin can synergistically inhibit the proliferation and survival of cisplatin-resistant HNSCC cells by inducing DNA damage and apoptosis, suggesting that Wee1 inhibition can improve the efficacy of cisplatin-resistant HNSCC (126). The results of a phase I clinical trial showed that the triplet combination of adavosertib (AZD1775), cisplatin, and docetaxel was safe and well tolerated, showing promising anti-tumor efficacy in patients with advanced HNSCC (127). In addition, phase I studies showed that adavosertib and cisplatin combined with radiotherapy enhanced the efficacy and safety of patients with TP53-mutated HNSCC (128, 129).

## Conclusions and perspectives

Several studies have demonstrated that the DDR system plays a crucial role in the occurrence, development, prognosis, and treatment of HNSCC. This review focuses on the possible DDR and repair pathway changes in HNSCC as well as the role of targeted DNA repair pathways in HNSCC therapy. Preclinical and clinical data suggest that the use of DDR inhibitors (PARP/ATM/ATR/DNA-PK/WEE1 inhibitors) alone or in combination with other therapies, such as radiotherapy or chemotherapy, is promising, which may provide a new direction for the treatment of HNSCC. However, the clinical application of targeted DNA repair therapy in HNSCC remains limited because of the lack of specific biomarkers. Thus, further investigations of the DDR mechanism in HNSCC are needed to identify possible early diagnostic biomarkers and therapeutic targets and to provide a theoretical basis for early diagnosis and targeted therapy of HNSCC.

## Author contributions

HL, YJ, and AH conceived the article. HL and NH wrote the manuscript. MR and NH helped to improve the manuscript. All authors contributed to the article and approved the submitted version.

## Funding

This study was supported by the National Natural Science Foundation of China (Grant Number: 81772870; 81472517; 82072983; 81777270). Shanghai Medical Garden Rising Star Talent Program [Hu [2021]. 99]. 2021 Youth Innovation Talent Introduction and Education Program of Shandong Province Universities (YJ).

## Conflict of interest

The authors declare that the research was conducted in the absence of any commercial or financial relationships that could be construed as a potential conflict of interest.

## Publisher’s note

All claims expressed in this article are solely those of the authors and do not necessarily represent those of their affiliated organizations, or those of the publisher, the editors and the reviewers. Any product that may be evaluated in this article, or claim that may be made by its manufacturer, is not guaranteed or endorsed by the publisher.
