# Supplementary material for: Targeting DNA damage response as a potential therapeutic strategy for head and neck squamous cell carcinoma
Source: Front Oncol. 2022 Oct 21;12:1031944. doi: 10.3389/fonc.2022.1031944 (PMC9634729; doi:10.3389/fonc.2022.1031944)
Supplement: Supplementary file 1 [file Table_1.docx]

Table S1. Clinical trials of PARP inhibitors combined with PD-1/PD-L1 inhibitors.

| **NCT Number** | **Combination** | **Other Interventions** | **Phase** | **Cancer** | **status** |
| --- | --- | --- | --- | --- | --- |
| NCT04191135 | Olaparib+Pembrolizumab | Carboplatin Gemcitabine | II | Locally Recurrent Inoperable or Metastatic Triple Negative Breast Cancer (TNBC) | Active, not recruiting |
| NCT03976323 | Olaparib+Pembrolizumab | Pemetrexed Carboplatin Cisplatin | III | Metastatic Nonsquamous Non-Small-Cell Lung Cancer (NSCLC) | Active, not recruiting |
| NCT03740165 | Olaparib+Pembrolizumab | Carboplatin Paclitaxel Bevacizumab Docetaxel | III | Advanced Epithelial Ovarian Cancer (EOC) Fallopian Tube Cancer Peritoneal Neoplasms | Active, not recruiting |
| NCT03834519 | Olaparib+Pembrolizumab | Abiraterone acetate Prednisone Enzalutamide | III | Metastatic Castration-resistant Prostate Cancer (mCRPC) | Active, not recruiting |
| NCT03976362 | Olaparib+Pembrolizumab | Carboplatin Paclitaxel Nab-paclitaxel | III | Metastatic Squamous Non-small Cell Lung Cancer (NSCLC) | Active, not recruiting |
| NCT04641728 | Olaparib+Pembrolizumab | _ | II | Recurrent or Metastatic Cervical Cancer Who Had Disease Progression During or After Platinum-based Chemotherapy | Active, not recruiting |
| NCT04209686 | Olaparib+Pembrolizumab | Paclitaxel | II | Previously Treated Advanced Gastric Adenocarcinoma | Recruiting |
| NCT04624204 | Olaparib+Pembrolizumab | Etoposide Platinum Radiotherapy | III | Newly Diagnosed Treatment-Naïve Limited-Stage Small Cell Lung Cancer (LS-SCLC) | Recruiting |
| NCT04683679 | Olaparib+Pembrolizumab | Radiotherapy | II | Metastatic Triple-Negative Breast Cancers | Recruiting |
| NCT04825990 | Olaparib+Pembrolizumab | _ | II | Recurrent/Metastatic, Platinum Resistant Nasopharyngeal Cancer | Recruiting |
| NCT04483544 | Olaparib+Pembrolizumab | _ | II | Advanced Cervical Cancer | Recruiting |
|  |  |  |  |  |  |
| NCT04633902 | Olaparib+Pembrolizumab | _ | II | Advanced Melanoma With Homologous Recombination (HR) Pathway Gene Mutation | Recruiting |
| NCT04753879 | Olaparib+Pembrolizumab | Nab-paclitaxel Gemcitabine Cisplatin Irinotecan Capecitabine | II | Untreated Metastatic Pancreatic Ductal Adenocarcinoma | Recruiting |
| NCT05268510 | Olaparib+Pembrolizumab | mFOLFOX-6 CapOX | II | Her-2 Negative Gastric/Gastroesophageal-junction (GEJ) Adenocarcinoma | Recruiting |
| NCT02861573 | Olaparib+Pembrolizumab | Docetaxel Prednisone Enzalutamide Dexamethasone Abiraterone acetate Lenvatinib Carboplatin Etoposide | I/II | Metastatic Castration-Resistant Prostate Cancer (mCRPC) | Recruiting |
| NCT05366166 | Olaparib+Pembrolizumab | Cisplatin Radiotherapy | II | Locally Advanced Head and Neck Squamous Cell Carcinoma (HNSCC) | Not yet recruiting |
| NCT05093231 | Olaparib+Pembrolizumab | _ | II | Metastatic Pancreatic Adenocarcinoma (PDA) | Not yet recruiting |
| NCT04592211 | Olaparib+Pembrolizumab | paclitaxel | I/II | Recurrent/Advanced Gastric and Gastro-esophageal Junction(GEJ) Cancer With HRR Mutation and MSS | Not yet recruiting |
| NCT05174832 | Olaparib+Pembrolizumab | Cisplatin Nab-paclitaxel | II | Triple-negative Metastatic Breast Cancer | Not yet recruiting |
| NCT05201612 | Olaparib+Pembrolizumab | _ | II | Homologous-recombination Deficient (HRD) Advanced Colorectal Cancer (CRC) | Not yet recruiting |
| NCT04380636 | Olaparib+Pembrolizumab+ Durvalumab | Etoposide Carboplatin Cisplatin Paclitaxel Pemetrexed Radiotherapy | III | Unresectable, Locally Advanced, Stage III Non-Small Cell Lung Cancer (NSCLC) | Recruiting |
| NCT02882308 | Olaparib+Durvalumab | Cisplatin | II | Histologically Proven Squamous Cell Carcinoma of the Head and Neck (SCCHN) Who Are Candidates for Surgery | Completed |
| NCT03951415 | Olaparib+Durvalumab | _ | II | Metastatic or Recurrent Endometrial Cancer | Active, not recruiting |
| NCT04015739 | Olaparib+Durvalumab | Bevacizumab | II | Advanced Epithelial Ovarian Cancer in Relapse | Active, not recruiting |
| NCT02734004 | Olaparib+Durvalumab | Bevacizumab | I/II | Advanced Solid Tumors | Active, not recruiting |
| NCT03810105 | Olaparib+Durvalumab | _ | II | Castration Sensitive Biochemically Recurrent Non-Metastatic Prostate Cancer Harboring Mutations in DNA Damage Repair | Active, not recruiting |
| NCT02953457 | Olaparib+Durvalumab | Tremelimumab | II | Recurrent Platinum Sensitive or Resistant or Refractory Epithelial Ovarian, Fallopian Tube, or Primary Peritoneal Cancer in Patients Who Carry a BRCA1 or BRCA2 Mutation | Active, not recruiting |
| NCT03851614 | Olaparib+Durvalumab | Cediranib | II | Mismatch Repair Proficient Colorectal Cancer Pancreatic Adenocarcinoma Leiomyosarcoma | Active, not recruiting |
| NCT02546661 | Olaparib+Durvalumab | AZD4547 AZD1775 Vistusertib AZD9150 Selumetinib | I | Muscle Invasive Bladder Cancer (MIBC) Who Have Progressed on Prior Treatment | Active, not recruiting |
| NCT02484404 | Olaparib+Durvalumab | Cediranib | I/II | Advanced Solid Tumors and Advanced or Recurrent Ovarian, Triple Negative Breast, Lung, Prostate and Colorectal Cancers | Recruiting |
| NCT03772561 | Olaparib+Durvalumab | AZD5363 | I | Advanced or Metastatic Solid Tumor Malignancies. | Recruiting |
| NCT03334617 | Olaparib+Durvalumab | AZD9150 AZD6738 Vistusertib Oleclumab trastuzumab deruxtecan | II | Non-Small Cell Lung Cancer (NSCLC) | Recruiting |
| NCT04739800 | Olaparib+Durvalumab | Paclitaxel Cediranib Maleate Pegylated Liposomal Doxorubicin Hydrochloride Topotecan Hydrochloride | II | Platinum-Resistant Recurrent Epithelial Ovarian Cancer, Primary Peritoneal or Fallopian Cancer Who Have Received Prior Bevacizumab | Recruiting |
| NCT03991832 | Olaparib+Durvalumab | _ | II | IDH-Mutated Solid Tumors | Recruiting |
| NCT03740893 | Olaparib+Durvalumab | AZD6738 | II | Neoadjuvant Chemotherapy Resistant Residual Triple Negative Breast Cancer | Recruiting |
| NCT04644289 | Olaparib+Durvalumab | _ | II | Prior to Primary Debulking Surgery in Histologically Proven High-grade Epithelial Ovarian Cancer (EOC) | Recruiting |
| NCT02849496 | Olaparib+Atezolizumab | _ | II | Homologous DNA Repair (HDR) Deficient, Locally Advanced or Metastatic Non-HER2-Positive Breast Cancer | Active, not recruiting |
| NCT04999605 | Olaparib+AK112 | _ | I/II | Recurrent Ovarian Cancer | Recruiting |
| NCT04475939 | Niraparib+Pembrolizumab | _ | III | Stage IIIB/IIIC or IV Non-Small Cell Lung Cancer | Recruiting |
| NCT02657889 | Niraparib+Pembrolizumab | _ | I/II | Advanced or Metastatic Triple-Negative Breast Cancer and Recurrent Ovarian Cancer | Completed |
| NCT03308942 | Niraparib+Pembrolizumab+ TSR-042 | _ | II | Locally Advanced and Metastatic Non-Small Cell Lung Cancer (NSCLC) | Completed |
| NCT03602859 | Niraparib+TSR-042 | paclitaxel bevacizumab | III | Stage III or IV Nonmucinous Epithelial Ovarian Cancer | Active, not recruiting |
| NCT03307785 | Niraparib+TSR-042 | Carboplatin-Paclitaxel Bevacizumab TSR-022 Carboplatin-Pemetrexed Carboplatin-Nab-Paclitaxel | I | Advanced or Metastatic Cancer | Active, not recruiting |
| NCT04681469 | Niraparib+TSR-042 | _ | II | HPV-negative Head and Neck Squamous Cell Carcinoma (HNSCC) | Recruiting |
| NCT04940637 | Niraparib+TSR-042 | _ | II | Advanced NSCLC and/or MPM, and Positive for PD-L1 Expression and Germline or Somatic Mutations in the HRR Genes | Recruiting |
| NCT04493060 | Niraparib+TSR-042 | _ | II | Germline or Somatic BRCA1/2 and PALB2-Related Pancreatic Cancer | Recruiting |
| NCT03574779 | Niraparib+TSR-042 | Bevacizumab Carboplatin Paclitaxel | I/II | Ovarian Cancer | Recruiting |
| NCT03651206 | Niraparib+TSR-042 | Pegylated liposomal doxorubicin Paclitaxel Topotecan Gemcitabine | II/III | Metastatic or Recurrent Endometrial or Ovarian Carcinosarcoma After at Least One Line of Chemotherapy | Recruiting |
| NCT04701307 | Niraparib+TSR-042 | _ | II | Small Cell Lung Cancer (SCLC) and Other High-Grade Neuroendocrine Carcinomas (NEC) | Recruiting |
| NCT04779151 | Niraparib+TSR-042 | _ | II | DNA Repair-deficient or Platinum-sensitive Solid Tumors | Recruiting |
| NCT03016338 | Niraparib+TSR-042 | _ | II | Recurrent Endometrial Cancer | Recruiting |
| NCT04544995 | Niraparib+TSR-042 | _ | I | Recurrent or Refractory Solid Tumors | Recruiting |
| NCT04837209 | Niraparib+TSR-042 | Radiotherapy | II | Metastatic, PD-L1 Negative or Immunotherapy-Refractory Triple-Negative Breast Cancer | Recruiting |
| NCT04584255 | Niraparib+TSR-042 | _ | II | BRCA-mutated Breast Cancer | Recruiting |
| NCT04673448 | Niraparib+TSR-042 | _ | I | BRCA-Mutated Breast, Pancreas or Ovary Cancer | Recruiting |
| NCT04679064 | Niraparib+TSR-042 | Pegylated liposomal doxorubicin Paclitaxel Gemcitabine Topotecan Bevacizumab | III | Recurrent, Ovarian, Fallopian Tube or Primary Peritoneal Cancer | Recruiting |
| NCT05065021 | Niraparib+TSR-042 | Bevacizumab Paclitaxel | II | Ovarian Cancer Fallopian Tube Cancer Primary Peritoneal Cancer | Not yet recruiting |
| NCT04508803 | Niraparib+HX008 | Trastuzumab Pyrrolitinib | II | GErm-line-mutAted Metastatic Breast Cancer | Recruiting |
| NCT04885413 | Niraparib+Sintilimab | _ | II | Recurrent/ Advanced Stage Endometrial Cancer | Recruiting |
| NCT03598270 | Niraparib+Atezolizumab | Carboplatin Paclitaxel Gemcitabine Pegylated liposomal doxorubicin (PLD) | III | Recurrent Ovarian, Tubal or Peritoneal Cancer and Platinum Treatment-free Interval (TFIp) >6 Months | Active, not recruiting |
| NCT04690855 | Talazoparib+Atezolizumab | Radiotherapy | II | gBRCA 1/2 Negative and PD-L1+ Metastatic Triple Negative Breast Cancer | Recruiting |
| NCT03964532 | Talazoparib+Avelumab | _ | I/II | Advanced Breast Cancer | Recruiting |
| NCT03330405 | Talazoparib+Avelumab | _ | II | Locally Advanced (Primary or Recurrent) or Metastatic Solid Tumors | Active, not recruiting |
| NCT03565991 | Talazoparib+Avelumab | _ | II | Locally Advanced or Metastatic Solid Tumors with BRCA or ATM Mutant | Active, not recruiting |
| NCT03694262 | Rucaparib+Atezolizumab | Bevacizumab | II | Previously Treated Recurrent and Progressive Endometrial Carcinoma | Active, not recruiting |
| NCT04276376 | Rucaparib+Atezolizumab | _ | II | DNA Repair-deficient or Platinum-sensitive Solid Tumors | Recruiting |
| NCT03522246 | Rucaparib+Nivolumab | _ | III | Epithelial Ovarian Cancer Primary Peritoneal Fallopian Tube Cancer | Active, not recruiting |
| NCT02873962 | Rucaparib+Nivolumab | Bevacizumab | II | Relapsed Epithelial Ovarian, Fallopian Tube or Peritoneal Cancer | Recruiting |
| NCT03639935 | Rucaparib+Nivolumab | _ | II | Advanced or Metastatic Biliary Tract Cancer | Recruiting |
| NCT03958045 | Rucaparib+Nivolumab | _ | II | Platinum-Sensitive Small Cell Lung Carcinoma | Recruiting |
| NCT02660034 | BGB-290+BGB-A317 | _ | I | Advanced Solid Tumors | Completed |
| NCT04978012 | Fluzoparib+Camrelizumab | _ | II | Recurrent/Metastatic Nasopharyngeal Carcinoma That Progressed After First-line Chemotherapy | Recruiting |
| NCT05392686 | Fluzopari capsule+Tirelizumab | _ | II | Advanced Non-small Cell Lung Cancer (Lung Squamous Cell Carcinoma) | Recruiting |
